# Genome-wide identification and expression analysis of the *SPL* transcription factor family and its response to abiotic stress in *Pisum sativum* L

**DOI:** 10.1186/s12864-024-10262-w

**Published:** 2024-05-31

**Authors:** Long Li, Jian bo Xu, Zhi wen Zhu, Rui Ma, Xiao zong Wu, Yu ke Geng

**Affiliations:** 1 Minzu University of China, 100010 Beijing, P.R. China; 2 School of Food and Biological engineering, Zhengzhou University of Light Industry, 450002 Zhengzhou, P.R. China; 3 College of Agronomy, Hebei Agricultural University, 071001 Baoding, P.R. China; 4 State Key Laboratory of Crop Stress Biology for Arid Areas, College of Plant Protection, Northwest A&F University, 712100 Yangling, Shaanxi P.R. China; 5 Zhengzhou University of Light Industry, 450002 Zhengzhou, P.R. China

**Keywords:** *Pisum sativum. L*, *SPL* genes, Genome-wide analysis, Growth and development, Abiotic stress, Plant hormone response, *PsSPL19*

## Abstract

Squamous promoter binding protein-like (*SPL*) genes encode plant-specific transcription factors (TFs) that play essential roles in modulating plant growth, development, and stress response. Pea (*Pisum sativum L.*) is a coarse grain crop of great importance in food production, biodiversity conservation and molecular genetic research, providing genetic information and nutritional resources for improving agricultural production and promoting human health. However, only limited researches on the structure and functions of *SPL* genes exist in pea (*PsSPLs*). In this study, we identified 22 *PsSPLs* and conducted a genome-wide analysis of their physical characteristics, chromosome distribution, gene structure, phylogenetic evolution and gene expression patterns. As a result, the *PsSPLs* were unevenly distributed on the seven chromosomes of pea and harbored the SBP domain, which is composed of approximately 76 amino acid residues. The phylogenetic analysis revealed that the *PsSPLs* clustered into eight subfamilies and showed high homology with *SPL* genes in soybean. Further analysis showed the presence of segmental duplications in the *PsSPLs*. The expression patterns of 22 *PsSPLs* at different tissues, developmental stages and under various stimulus conditions were evaluated by qRT-PCR method. It was found that the expression patterns of *PsSPLs* from the same subfamily were similar in different tissues, the transcripts of most *PsSPLs* reached the maximum peak value at 14 days after anthesis in the pod. Abiotic stresses can cause significantly up-regulated *PsSPL19* expression with spatiotemporal specificity, in addition, four plant hormones can cause the up-regulated expression of most *PsSPLs* including *PsSPL19* in a time-dependent manner. Therefore, *PsSPL19* could be a key candidate gene for signal transduction during pea growth and development, pod formation, abiotic stress and plant hormone response. Our findings should provide insights for the elucidating of development regulation mechanism and breeding for resistance to abiotic stress pea.

**Supplementary Information:**

The online version contains supplementary material available at 10.1186/s12864-024-10262-w.

## Background

Pea (*Pisum sativum****L.***) is a legume plant and a member of the genus *Pisum*. It is an annual climbing plant and an important source of food [1, 2]. *Pisum sativum* is a source of high-quality proteins and other nutrients such as dietary fiber, vitamin C, vitamin K, and minerals [3, 4]. Additionally, pea is used to produce green manure, which improves soil quality by fixing nitrogen and providing nutrients to crops [5, 6]. Meanwhile, the strong drought - and cold - resistant properties made pea adaptable to a wide range of climates [7].

Transcription factors (TFs) are a class of proteins that regulate gene transcription. They regulate gene expression by binding to specific regions in gene promoters, activating or inhibiting gene transcription, and thus regulate a variety of biological processes, such as plant growth, development, differentiation and response to environmental stimuli [8, 9]. Squamosa promoter binding protein-like (SPL) TFs are a ubiquitous family of TFs in various plants and contain a conserved SBP domain, two Cys2/His2 type zinc fingers, and two types of zinc fingers which is a structurally stable zinc finger motif formed by two cysteines (Cys) and two histidines (His) via ring and helical connections, and this domain binds to specific regions of DNA [10–12]. SPL is usually involved in plant growth, flower organ development and fruit development [10, 13]. In order to explore the biological functions and regulatory mechanisms of *SPLs* in plant growth, development and stress response, various plant species have been studied and elaborated by scholars, such as *Arabidopsis thaliana* [14, 15], *Morus alba* [16], *Nicotiana tabacum* [17], *Zea mays* [18], *Setaria italica* [19], *Vaccinium corymbosum* [20], *Vitis vinifera* [21], *Fagopyrum tataricum* [22], *Solanum lycopersicum* [23], *Codonopsis pilosula* [23], *Triticum aestivum* [24], and *Jatropha curcas* [25].

SPL TFs were first identified in Snapdragon (*Antirrhinum majus L*.). AmSPL regulates the formation and development of flower organs via binding to promoters of genes related to flower development [26]. AtSPL9 and AtSPL15 in *Arabidopsis thaliana* are highly expressed in stem apex meristem and participate in leaf differentiation to regulate plant leaf morphology and structure [27, 28]. Cui et al. confirmed that *AtSPL9* is negatively regulated by miR156, which promotes early flowering of *Arabidopsis thaliana* under salt and drought stress. In addition, AtSPL9 can compete with TT8 (Transparent testa8) combined with PRODUCTION OFANTHOCYANIN PIGMENT 1 (PAP1) [29], such competition interferes with the stability of MYB-bHLH-WD40 (MBW) transcription-activating complex, directly prevents the expression of flavonoid synthesis gene dihydroflavonol 4-reductase (DFR), and thus negatively regulates anthocyanin accumulation [15]. *AtSPL3* and *AtSPL4* are significantly differentially expressed in the apical meristem of *Arabidopsis thaliana*, but both are involved in the regulation of flowering [15, 30]. Loss-of-function mutants in *OsSPL14*, *OsSPL16* and *OsSPL18* lead to flower dysplasia, including increased inflorescence branching, atypical flower morphology and anther development defects [31]. This implies that these SPL TFs play essential regulatory roles in the formation and development of rice flower organs.

In this study, we identified *PsSPLs* from pea genome sequence obtained from publicly available databases using bioinformatics analysis. A genome-level comprehensive analysis was performed to explore their structural characteristics, motif composition, chromosome localization and evolutionary relationships. Then, we used real-time quantitative fluorescent PCR (qRT-PCR) to analyze the expression patterns of *PsSPLs* of different tissues, expression changes during pod formation stage, induced expression level under abiotic stress and hormone response, separately. The results provide an experimental basis for further revealing the mechanism of pea *SPL* gene family in regulating the growth and development, yield and environmental response, which possesses important application value for molecular breeding of pea with high yield and quality.

## Methods

### Gene identification

The whole-genome sequence of *Pisum sativum* was retrieved from the Ensembl Genomes website (http://ensemblgenomes.org/), whereas the whole-genome sequence of *Arabidopsis thaliana* was downloaded from The *Arabidopsis* Information Resource (TAIR) website (https://www.arabidopsis.org/). BLASTp [32](with a score cutoff of ≥ 100 and an e-value cutoff of ≤ 1e-10) was used to align *AtSPL* with the complete genome sequence of closely related species rye to identify candidate *SPL* genes. Subsequently, the hidden Markov model (HMM) of the SBP conserved domain was retrieved from the Pfam database (http://pfam.sanger.ac.uk/), and HMMER 3 (http://plants.ensembl) was used to identify non-redundant *SPL* genes [33]. The selected *SPL* genes were confirmed using the SMART tool (http://smart.embl-heidelberg.de/) [34, 35]. A total of 22 *SPL* genes were obtained for the subsequent analyses. ExPasy tool was used to determine the physical characteristics of the genes, including sequence length, isoelectric point (pI), and molecular weight (MW). The WoLFPSORT tool (https://wolfpsort.hgc.jp/) was used to predict the subcellular localization of the selected *SPL* genes.

### Gene structure, chromosomal distribution and gene duplication analysis and classification of the *PsSPLs*

The MEME website (http:/meme.nbcr.net/meme/intro.html) was used to identify the motifs in *PsSPLs*, with a maximum of 10 motifs set for the search. The GSDS (http://gsds.cbi.pku.edu.cn) tool was used to analyze the distribution of introns and exons in the sequences of *PsSPLs*. The Circos website was used to identify the location of *PsSPLs* in chromosomes. MCScanX toolkit was used to evaluate the collinear duplication events on the chromosomes [36]. Mega7.0 software was used to perform multiple sequence comparison of *SPL* genes from pea and *Arabidopsis thaliana*, and the phylogenetic analysis of different *SPL* genes was performed by neighborhood linkage (NJ) method and phylogenetic tree construction [37]. At the same time, the NJ method was also used for phylogenetic analysis of *SPL* genes from Pea, *Oryza sativa*, *Zea mays*, *Hordeum vulgare*, *Arabidopsis thaliana*, *Triticum aestivum* (hexaploid) and *Aegilops tauschii Coss*, Moreover, the evolutionary relationships among different species and subfamilies are further discussed.

### Evaluation of varying growth conditions and the effect of different hormone treatments on *Pisum sativum*

In this study, pea No. 6 cultivar provided by Zhengzhou University of Light Industry was taken as the research material. Fully mature pea seeds were selected and evenly sown in a greenhouse under the following growth conditions: 16 h light at 28 °C / 8 h darkness at 20 °C, and 75% constant relative humidity. The Tissues (roots, stems, leaves, anthers and styles, seeds) of pea plants with the same growth state at 21-day growth are collected and stored in an ultra-low temperature refrigerator of -80 °C. Parallelly, Pea plants were treated with four abiotic stresses, including salt stress (5% sodium chloride), low temperature stress (4 °C), drought stress (30% PEG 6000) and high temperature stress (40 °C) for 0, 1, 4 and 12 h, respectively, and the untreated plants obtained at the same time point were used as controls, the whole plants were collected and stored in an ultra-low temperature. After that, the pea plants at the filling stage were treated with ABA, IAA, JA, SA and GA3 at concentrations of 100µM for 0, 1, 4 and 12 h, respectively and the whole plants were sampled and preserved at ultra-low temperature. In the above experiments, three plants were selected for one type of treatment, and three independent biological replicates were set up for each.

### Total RNA extraction, cDNA synthesis, and qRT-PCR analysis

RNA extraction kit (Tiangen Biotech, Beijing, China) was used to extract RNA from different tissue samples collected. HiScript Q RT Super Mix for qPCR (Vazyme #R122) was used for reverse transcription to obtain cDNA, and AceQ qPCR SYBR Green Master Mix was used for qRT-PCR analysis of SPL gene. Primers for SPL gene qRT-PCR experiments were designed using Primer5.0 software (Additional file 5: Table S5). In this study, the glyceraldehyde-3-phosphate dehydrogenase (GADPH) gene was used as an internal reference for gene expression quantification. The experiment was repeated three times and the relative gene expression was calculated by 2^-(ΔΔCt) method. Compared with the control group, we defined a significant difference in expression levels as ≥ 2-fold or ≤ 0.5-fold [38].

### Statistical analysis

In this study, all data were subjected to analysis of variance (ANOVA) using the JMP 6.0 software (SAS Institute). The least significant difference (LSD) test was performed to determine the significance of differences between different treatment groups [38, 39]. A significance level (e.g., 0.05 or 0.01) is typically set in the LSD test to determine if the differences are statistically significant. Origin version 8.0 (OriginLab, Northampton, MA, USA) was used to generate the histograms for gene expression levels.

## Results

### Identification of *SPL* genes in Pea

A comprehensive and systematic analysis of the *PsSPLs* was conducted, leading to identification and characterization of 22 *PsSPLs*. The length of proteins encoded by these genes ranged from 139 (PsSPL3) to 1025 (PsSPL9) amino acids. The maximum molecular weight of the proteins encoded by *PsSPL* was 113.918 kDa (*PsSPL18*), whereas the minimum molecular weight was15.9 kDa (*PsSPL4*). The pI values ranged from 6 to 9.28 (*PsSPL7* and *PsSPL12*), with an average value of 7.622. All the *PsSPLs* harbored the conserved SBP domain, and four of them contain ANK (ankyrin) domain (Additional file 1: Table S1). The ANK domain is a conserved domain involved in protein-protein interaction and it plays an important role in various biological processes, including cell signaling, protein interactions and assembly of protein complexes. Subcellular localization prediction revealed that all the PsSPLs were localized in the nucleus, however. some were observed in different cellular tissues with 9 SPLs localized in the endoplasmic reticulum, 6 SPLs in the chloroplasts and seven in the cytoplasm (Additional file 1: Table S1).

### *PsSPLs* exhibit high evolutionary conservation

**Fig. 1:**
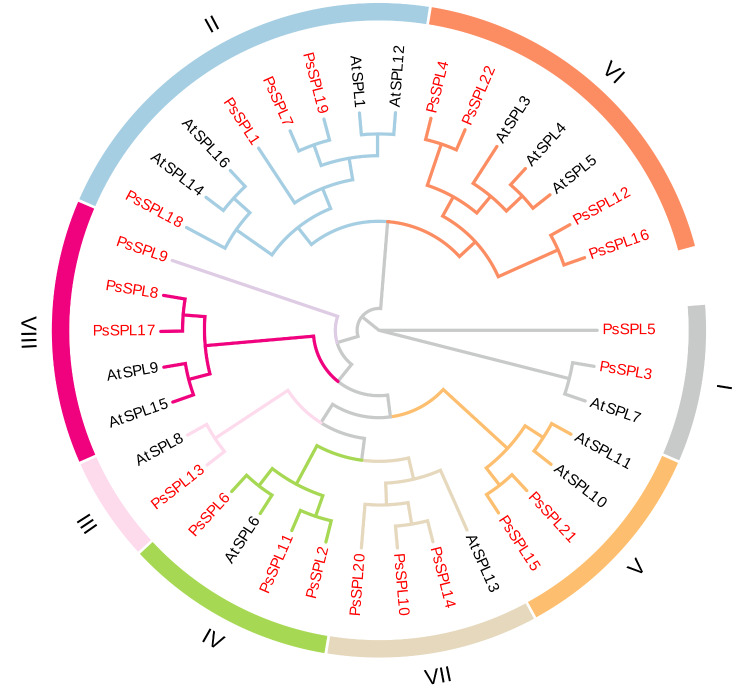
Phylogenetic analysis of SPL proteins in pea and *Arabidopsis thaliana*. 22 PsSPLs were divided into eight clades (I–IX) and indicated with different colors. The red pentacle represents Pea, the black circle represents *Arabidopsis thaliana*

Phylogenetic analysis of 22 *PsSPLs* from pea and 16 *AtSPLs* from *Arabidopsis thaliana* was conducted in this study. The *PsSPLs* formed three branches (Group 1–8) based on the classification method proposed by Cenci and Rouard consistent with the classification of SPL proteins in *Arabidopsis thaliana* [40]. This finding indicated conservation of *SPLs* across the evolutionary process without any loss events. In addition, we observed an uneven distribution of *SPLs* exist among the eight subfamilies. Subfamilies II and VI contained the largest number of members (4 *PsSPLs*), whereas subfamily III possessed the lowest number of genes (only 1 *PsSPL*) (Fig. 1). Subfamilies VII, VIII, and IV owned the same number of *SPL*s (3 *PsSPLs*). Subfamilies I and V each contain 2 *PsSPLs* (Fig. 1; Additional file 1: Table S1). Comparative analysis of the phylogenetic tree revealed that *PsSPL* clustered with *AtSPL* (bootstrap support ≥ 70), implying that these proteins may exert similar biological functions. These findings would provide a basis for further exploring the evolution and function of the *SPL* gene family in plants.

### Conserved motifs and structure analysis of the *PsSPLs*

**Fig. 2:**
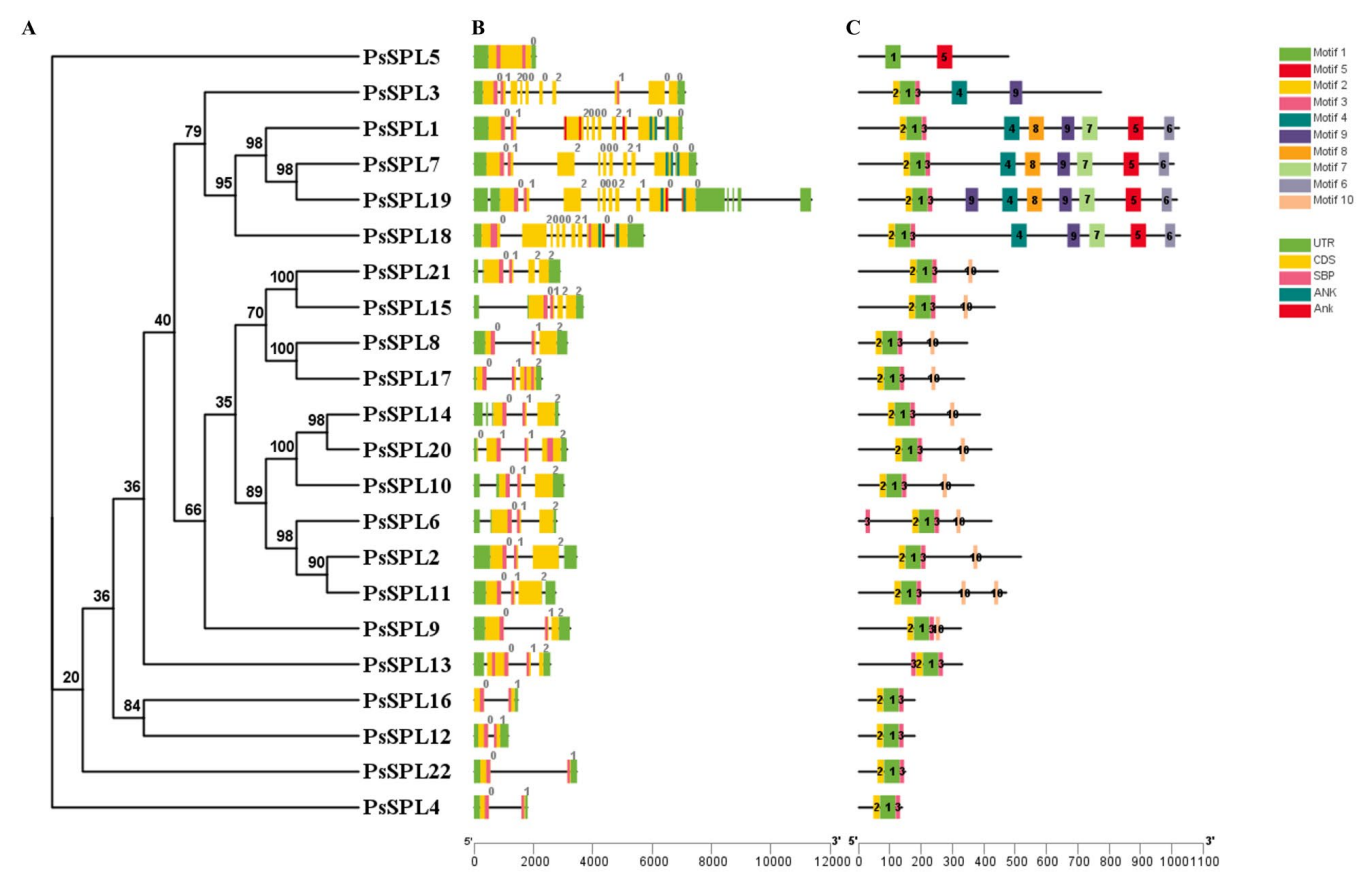
Phylogenetic relationships, gene structure, and motif distributions of *PsSPL* genes. Phylogenetic tree was constructed for each node with 1000 replicates using the NJ method (**A**). Exons and introns are indicated by yellow rectangles and grey lines, respectively (**B**). These numbers indicate the annotation file of the different phases of gene CDS, which are defined as “0”, “1”, and “2”. C Amino acid motifs in the SPL proteins (1–10) were represented by colored boxes, the black lines indicate relative protein lengths

Comparing the exons and introns of genes provided a comprehensive understanding of gene structure, function, and regulation in an organism [41, 42]. The results showed that different genes exhibited varying numbers of exons and introns, ranging from 0 to 14. *PsSPL19* exhibited the highest number of introns (14), whereas *PsSPL5* lacked intronic structures (Fig. 2). *PsSPL1*, *PsSPL7*, *PsSPL18*, and *PsSPL19*, members of subfamily II, exhibited the most complex gene structures, indicating that subfamily II may have different functions. Typically, members of the same gene family exhibit conserved intron and exon structures. This conservation can be attributed to the conserved structure of introns and exons caused by gene replication and recombination during the evolution of gene family members.

We identified 10 conserved motifs conserved across the genomic sequences of different genes using the MEME tool [43, 44], indicating the homology and conservation of regions in these genes. We named these motifs as Motif 1–10. The different subfamilies exhibited some differences in motifs, but all genes had Motif 1, indicating that Motif 1 is a conserved domain in SPL genes (Fig. 2). Subfamily II exhibited 9 of the 10 motifs identified in this study (lacked Motif 10). All genes exhibited Motif 1, Motif 2, and Motif 3. Subfamilies IV, V, VII, and VIII had Motif 10, which was absent in the other subfamilies. Motif 2 was located at the beginning of all patterns, whereas Motif 1 was positioned between Motif 2 and Motif 3 (Fig. 2; Additional file 2: Table S2). The conservation of motifs implied that the subfamily potentially emerged through evolutionary processes such as gene duplication and recombination. These findings indicate that the genes may form clusters in the genome with similar structural and functional features.

### Chromosomal distribution and gene duplication of *PsSPLs*

**Fig. 3:**
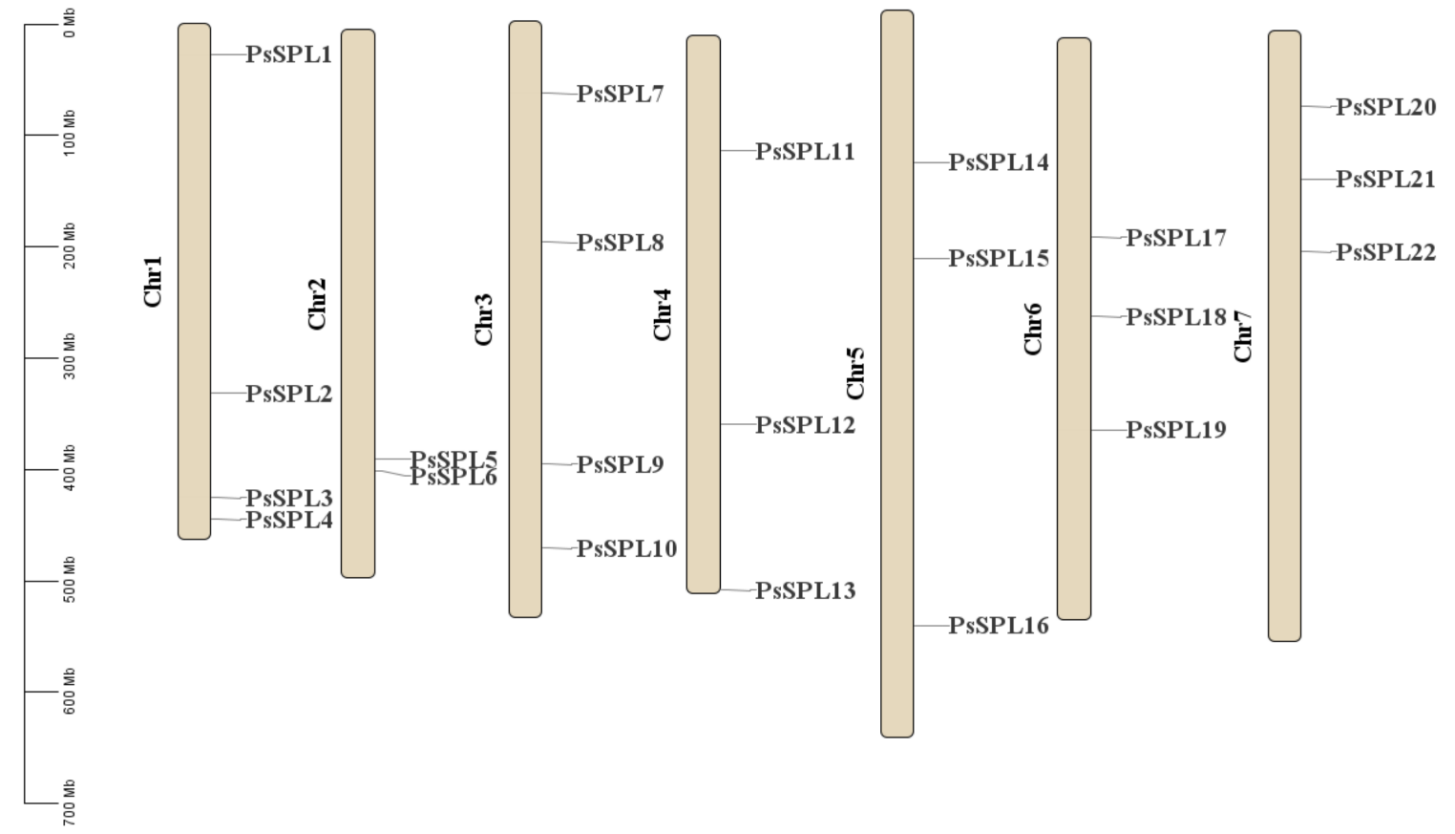
Schematic representation of the chromosomal distribution of *PsSPLs*. Vertical bars represent the chromosomes of Pea. The chromosome number is indicated to the left of each chromosome. The scale on the left represents chromosome length

The SPL genes were mapped to different chromosomes using the latest version of the Pea genome database [45]. The 22 *PsSPLs* were unevenly distributed across seven chromosomes (Fig. 3). Chr1 and Chr3 harbored the highest number of *PsSPL*s (4 genes, approximately 18.18%), followed by Chr4, Chr5, Chr6, and Chr7, which each contained three *SPLs* (approximately 13.63% each). Chr2 harbored the least number of *PsSPLs* (2 genes, approximately 9.09%). The *PsSPLs* are randomly distributed across different chromosomes with no significant correlation between the distribution of *SPLs* on chromosomes to their function and structure.

**Fig. 4:**
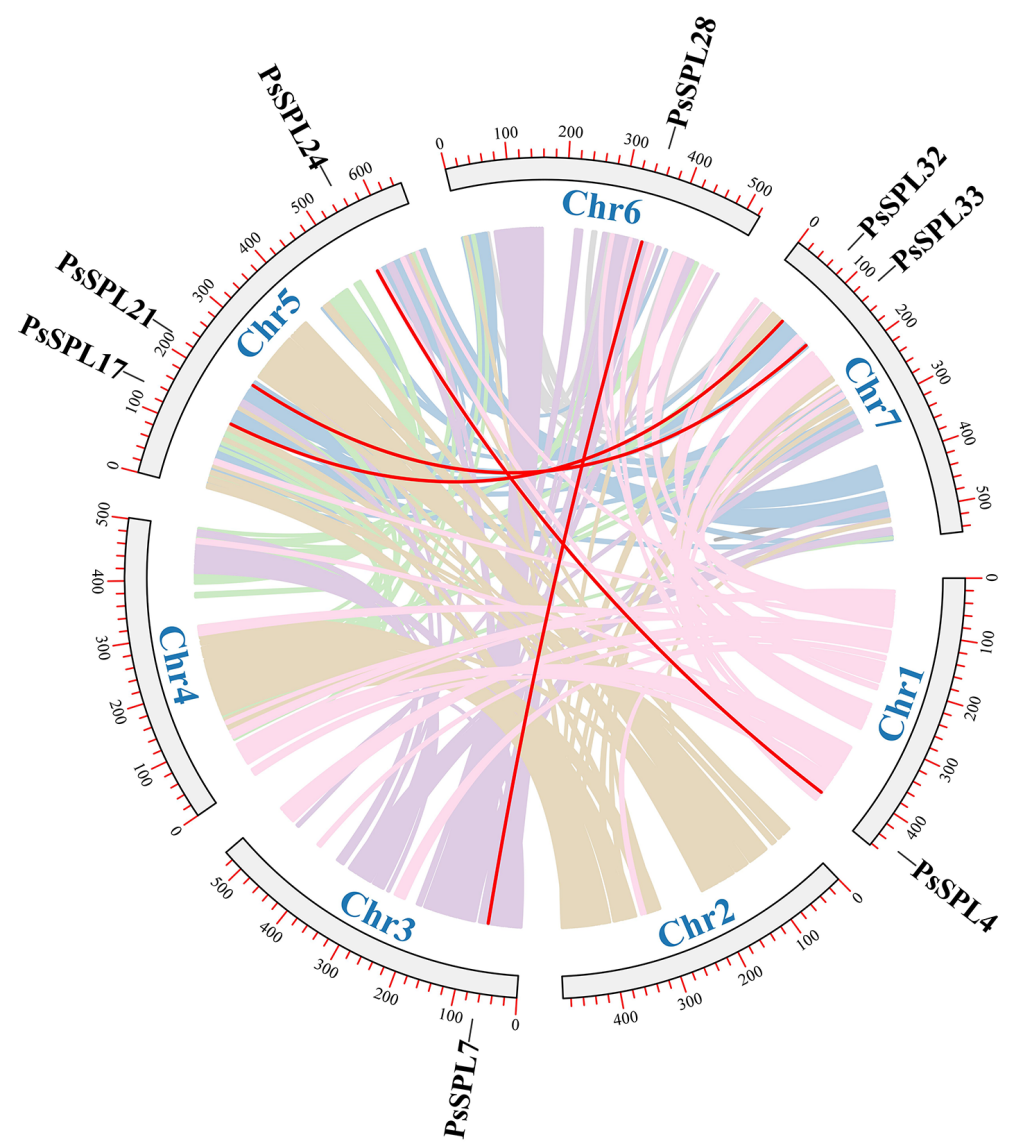
Schematic representation of the chromosomal distribution and interchromosomal relationships of *PsSPLs*. Colored lines denote all synteny blocks in the Pea genome, and the red lines denote duplicated *SPL* pairs. The chromosome number is denoted at the bottom of each chromosome

Tandem duplication events and segmental duplication events are key forms of gene duplication processes, which contribute to genomic diversity and play important roles in shaping the structure and function of the genome [46, 47]. In this study, no any tandem duplication event was detected on the chromosomes (Fig. 4; Additional file 3: Table S3). However, we found eight homologous sequences produced by four pairs of fragments distributed on five chromosomes, this indicated the existence of evolutionary relationships among *PsSPLs*, Chr5 contains the largest number of *PsSPLs* (*n* = 3), followed by Chr 7 (*n* = 2), while Chr 3, Chr 4, and Chr 6 each contains only one *PsSPL* (Fig. 4).

### *PsSPLs* are evolutionarily related to *SPLs* in other species

**Fig. 5:**
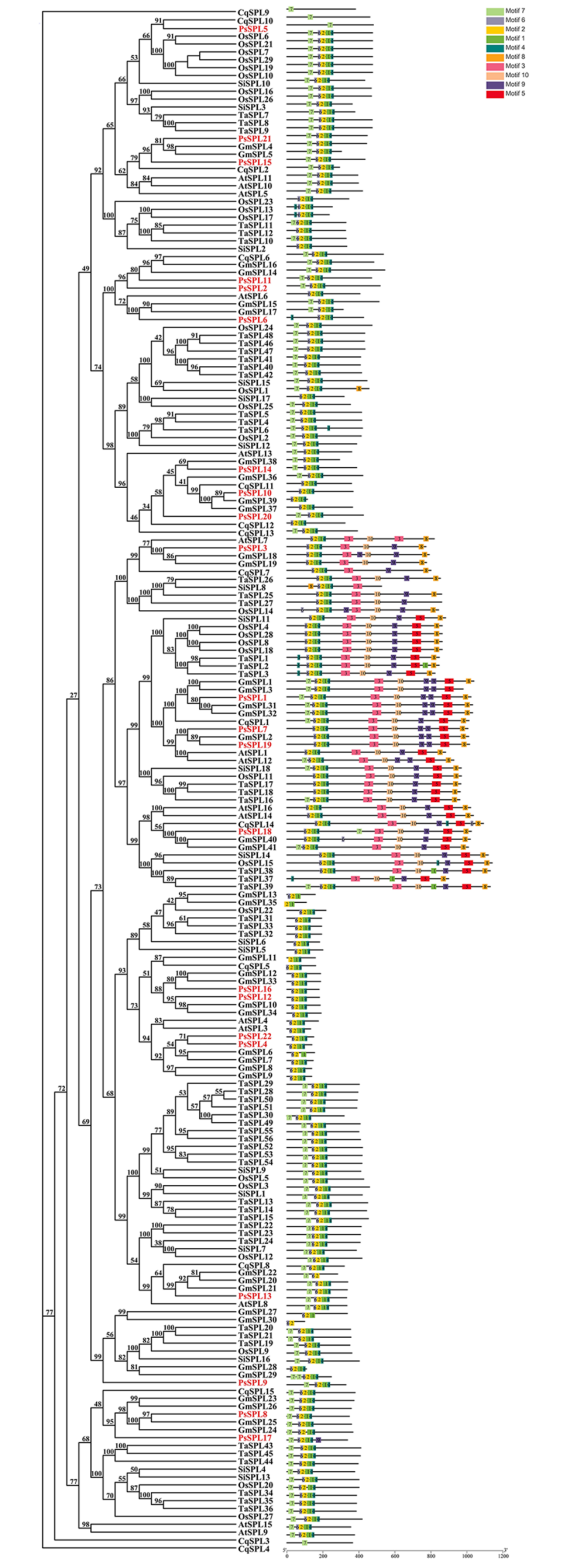
Phylogenetic relationships and motif compositions of the PsSPLs with six different plant species (*Arabidopsis thaliana*, *Solanum lycopersicum*, *Vitis vinifera*, *Sorghum bicolor Moench*, *Oryza sativa* and *Zea mays*). Outer panel: an unrooted phylogenetic tree constructed using Geneious R11 with the neighbor-joining method. Inner panel: distribution of conserved motifs in SPL proteins. The differently colored boxes represent different motifs and their positions in each SPL protein sequence. The sequence information for each motif is provided in Additional file 2 (Table S2)

We compared the *PsSPL* gene with *SPL* genes from two dicot plants (*Arabidopsis thaliana* and *Glycine max*) and four monocot plants (*Setaria italica*, *Triticum aestivum*, *Oryza sativa* and *Chenopodium quinoa*) to evaluate the genomic diversity among different species and explore the common ancestors and evolutionary relationships. We constructed a phylogenetic tree using the NJ method based on 10 conserved motifs identified in the genes, obvious distinctions in motifs among the different species were observed, but the same subfamily exhibited motif conservation (Fig. 5). Most genes harbored Motif 1, Motif 2, and Motif 4. Subfamily II exhibited all the 10 motifs, implying that this subfamily may have a more complex and diverse gene regulatory network compared with the other subfamilies. All members of the subfamily except for subfamily VI started with Motif7. Most subfamilies exhibited high homology with the Gm*SPL* gene family of soybean, indicating that pea and soybean may share a common ancestor or had high degree of structural and sequence conservation during evolution.

We conducted a collinearity analysis based on the genes of *Pisum sativum* and six representative species to elucidate the evolutionary relationships and functional conservation across different species. The results revealed collinearity between 22 *PsSPL* genes and genes from ***A****rabidopsis thaliana* (15), *Setaria italica* (18), *Chenopodium quinoa* (23), *Glycine max* (29), *Oryza sativa* (29), and *Triticum aestivum* (56). Monocot plants and pea exhibited a closer evolutionary relationship than the relationship between dicot and monocot plants. We identified 52 gene pairs shared between ***Glycine max*** and *Pisum sativum*, 15 gene pairs shared with *Arabidopsis thaliana*, 7 gene pairs shared with *Oryza sativa*, 7 gene pairs shared with *Setaria italica*, 6 gene pairs shared with *Triticum aestivum*, and 6 gene pairs shared with *Chenopodium quinoa*.

**Fig. 6:**
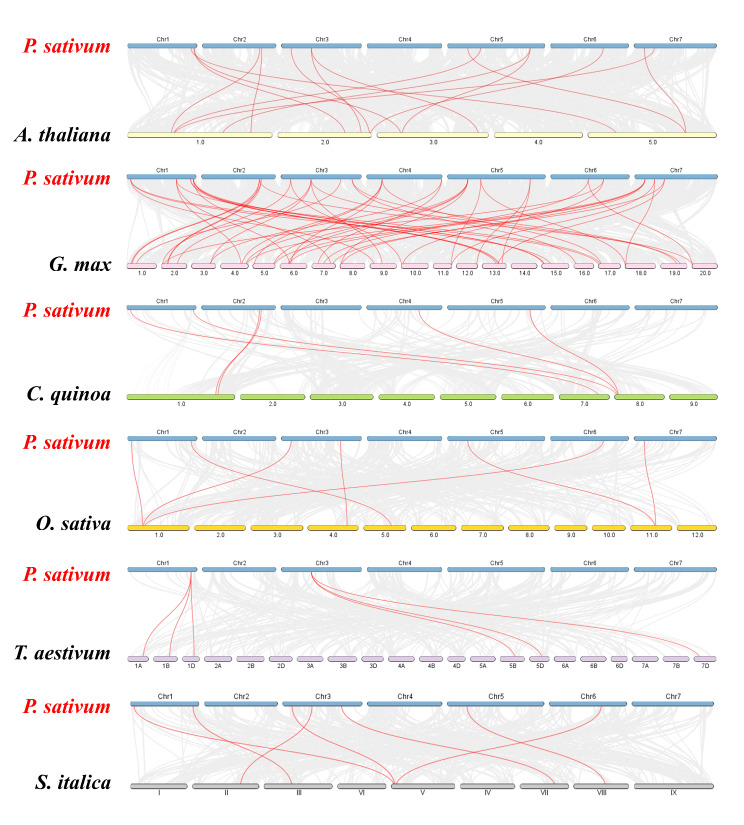
Synteny analyses of the *SPL* genes between *Pisum sativum* and six representative plant species (*Arabidopsis thaliana*, *Glycine max*, *Chenopodium quinoa*, *Oryza sativa, Triticum aestivum* and *Setaria italica*). Gray lines on the background indicate the collinear blocks in *Pisum sativum* and other plant genomes; red lines highlight the syntenic of *SPL* gene pairs in *Pisum sativum*

Comparative analysis of collinearity among the six plants revealed that the number of collinear *SPL* genes between *Pisum sativum* and *Triticum aestivum* and *Chenopodium quinoa* was the lowest, whereas the number of collinear *SPL* genes between *Pisum sativum* and *Glycine max* was the highest. Interestingly, these species shared some common collinear genes. For example, *PsSPL3* exhibited collinearity with AT5G18830.3 / Os05t0408200-01 / TraesCS1A02G255300.1 / KQL15519. This finding indicates that these collinear genes are highly conserved and possibly existed before the divergence of these species (Fig. 6; Additional file 4: Table S4). Collinear genes are used to explore genomic similarities and differences, evolutionary relationships, and functional conservation among different species. The findings on collinear genes provide information on genetic variations and evolutionary relationships among different species.

### Expression patterns of the *PsSPL*s in different plant organs

**Fig. 7:**
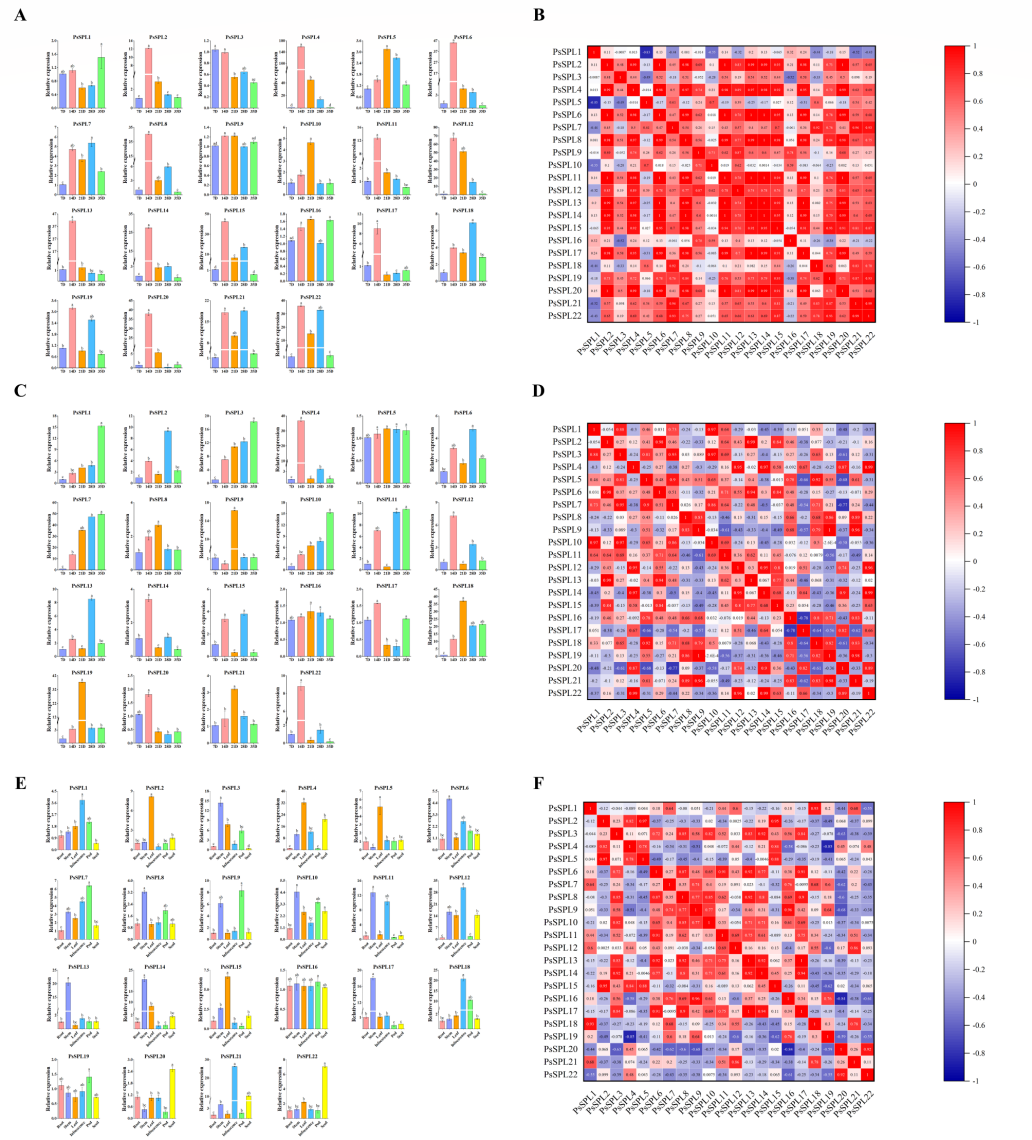
Tissue-specific gene expression of 22 *PsSPLs* and gene expression during fruit development. Expression patterns of 22 *PsSPLs* and in fruit of different stage and flower, leaf, root, stem were detected by qPCR. Error bars are obtained from three measurements. Lowercase letters indicate significant differences between treatments (*α* = 0.05, LSD) (**A**, **C**, **E**). Coexpression analysis of 22 *PsSPLs* (**B**, **D**, **F**)

In order to evaluate the potential function of *PsSPLs*, qRT-PCR was used to analyze the expression of 22 *PsSPLs* in four organs: root, stem, leaf, flower and fruit. The results show that *PsSPLs* were expressed differently in these four organs, which reflects the specific biological functions and adaptation strategies of plants at different growth and development stages and under different environmental conditions. Three genes (*PsSPL3*, *PsSPL7*, and *PsSPL19*) were most expressed in stems, while eight genes (*PsSPL2*, *PsSPL5*, *PsSPL6*, *PsSPL9*, *PsSPL11*, *PsSPL14*, *PsSPL15*, and *PsSPL20*) were most expressed in leaves. *PsSPL1*, *PsSPL12*, *PsSPL18* and *PsSPL20* were highly expressed in flowers (Fig. 7). Genes from the same subfamily may have maintained similar expression patterns over the course of evolution, reflecting that they may share a common ancestor and have maintained similar regulatory patterns over the course of evolution. It is obvious that the expression levels of all *PsSPLs* in roots are lower than those in stems, leaves and flowers, so we speculate that *SPLs* may be closely related to the development of stems, leaves and flowers of plants.

Previous studies have shown that the expression level and pattern of *SPL* during fruit development may be related to biological processes such as fruit growth, ripening and storage. Therefore, we selected 15 representative genes for qRT-PCR verification at five post-anthesis stages (7DPA, 14DPA, 21DPA, 28DPA and 35DPA). As shown in Fig. 7, almost all genes showed differential expression patterns at different time periods. In pea fruits, we found that *PsSPL4* had the most significant differential expression at 14DPA stage, while *PsSPL3* showed a downward trend with the increase of time. Interestingly, *PsSPL9* remained stable over time, suggesting that it may not be associated with fruit development. In addition, we found that most of the significantly differentially expressed genes (DEGs) were concentrated at 14DPA stage, such as *PsSPL2*, *PsSPL4*, *PsSPL6*, *PsSPL8*, *PsSPL11*, *PsSPL12*, *PsSPL13*, *PsSPL14*, *PsSPL15*, *PsSPL17*, *PsSPL2*, *PsSPL4*, *PsSPL6*, *PsSPL8*. *PsSPL19* and *PsSPL22*. These results indicated that the expression of most *PsSPLs* were mainly concentrated in the early stage of pea fruit. The expression level of *PsSPL1* reached its maximum value at 35DPA stage, which may be related to the later stage of fruit development. In addition, we found that the expression of *PsSPL7* was the highest in the pod, and most of the genes showed significant differential expression. The expression of *PsSPL5* and *PsSPL16* was not significantly different at different times, indicating that it was not related to pod development of pea. The heat map showed that there was a correlation between different *PsSPL*s, and most *PsSPLs* were positively correlated. However, some *PsSPLs* were negatively correlated, such as *PsSPL6* and *PsSPL21*/*PsSPL1*, and *PsSPL1* and *PsSPL9* (*P* < 0.05).

### Expression patterns of *PsSPLs* under various abiotic stress conditions

**Fig. 8:**
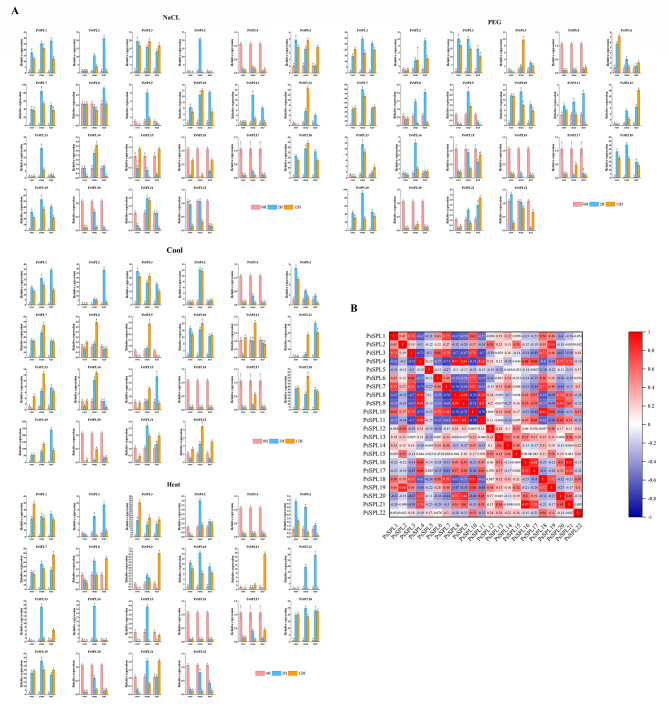
Expression of *PsSPLs* in plants subjected to abiotic stresses (PEG, NaCl, heat, and cool treatments) at the seedling stage in three organs (root, stem, and leaf). (**A**) Changes in expression of representative genes analyzed by qRT-PCR. Error bars were obtained from three measurements. The lowercase letter above the bar indicates a significant difference (α = 0.05, LSD) among the treatments. (**B**) Coexpression analysis of 22 *PsSPL*s in several plant organs. Positive numbers: positive correlations; negative numbers: negative correlations. Red numbers indicate a significant correlation at the 0.05 level

Prediction of gene function by exposing plants to various stress treatments can offer valuable insights into elucidating the biological functions of genes under adverse conditions, providing information on plant adaptation mechanisms and regulatory networks in response to stress [48]. We used four stress treatments to explore the effects of various stress conditions on the expression patterns of *PsSPL* genes in different tissues. The results showed that various stress conditions induced differential expression of *PsSPL* genes in different tissues and at different time points. *PsSPL* genes are implicated in stress signaling pathways, so their expression levels are upregulated with increase in treatment time, indicating their important regulatory role in alleviating stress in plants. The expression of most *SPL* genes was upregulated in stems under cold stress conditions. *PsSPL1*, *PsSPL3*, *PsSPL7*, *PsSPL10*, *PsSPL18*, and *PsSPL19* genes exhibited significant differential expression in roots, leaves, and stems, with upregulation at the early stages of stress treatment and then downregulation at the late stages. *PsSPL19* had the highest expression level under PEG and salt stress condition, and the expression of the other genes was also upregulated under these two stress treatments. Conversely, the expression of *PsSPL5*, *PsSPL15*, *PsSPL16*, *PsSPL17*, and *PsSPL20* were downregulated under PEG and salt stress conditions. Tissue-specific responses were mainly observed in stems under cold and heat stress treatments. *PsSPL4*, *PsSPL10*, *PsSPL13*, *PsSPL14* and *PsSPL18* genes displayed similar expression patterns, with increases expression levels in different tissues observed over treatment time. On the contrary, the expression of *PsSPL5*, PsSP16 and *PsSPL20* was downregulated over time. The genes showed different expression patterns under different treatments, and the significant downregulation observed at the start of stress induction may be attributed to the rapid regulation and adaptation process of genes to the treatments. *PsSPL19* expression was upregulated under the four stress treatments in different tissues, indicating its potentially important role in stress responses. We performed correlation analysis and generated a heatmap to show the correlation in expression levels among PsSPL members under stress. The results showed that the expression levels of *PsSPL2*, *PsSPL3*, *PsSPL6*, *PsSPL9*, *PsSPL11*, *PsSPL12*, *PsSPL14 PsSPL15*, *PsSPL18*, and *PsSPL19*, genes were significantly positively correlated, whereas the expression of *PsSPL1* was significantly positively correlated with *PsSPL5* expression (Fig. 8). The expression of some *PsSPL* genes was significantly negatively correlated with the expression of *PsSPL5* and *PsSPL20*.

**Fig. 9:**
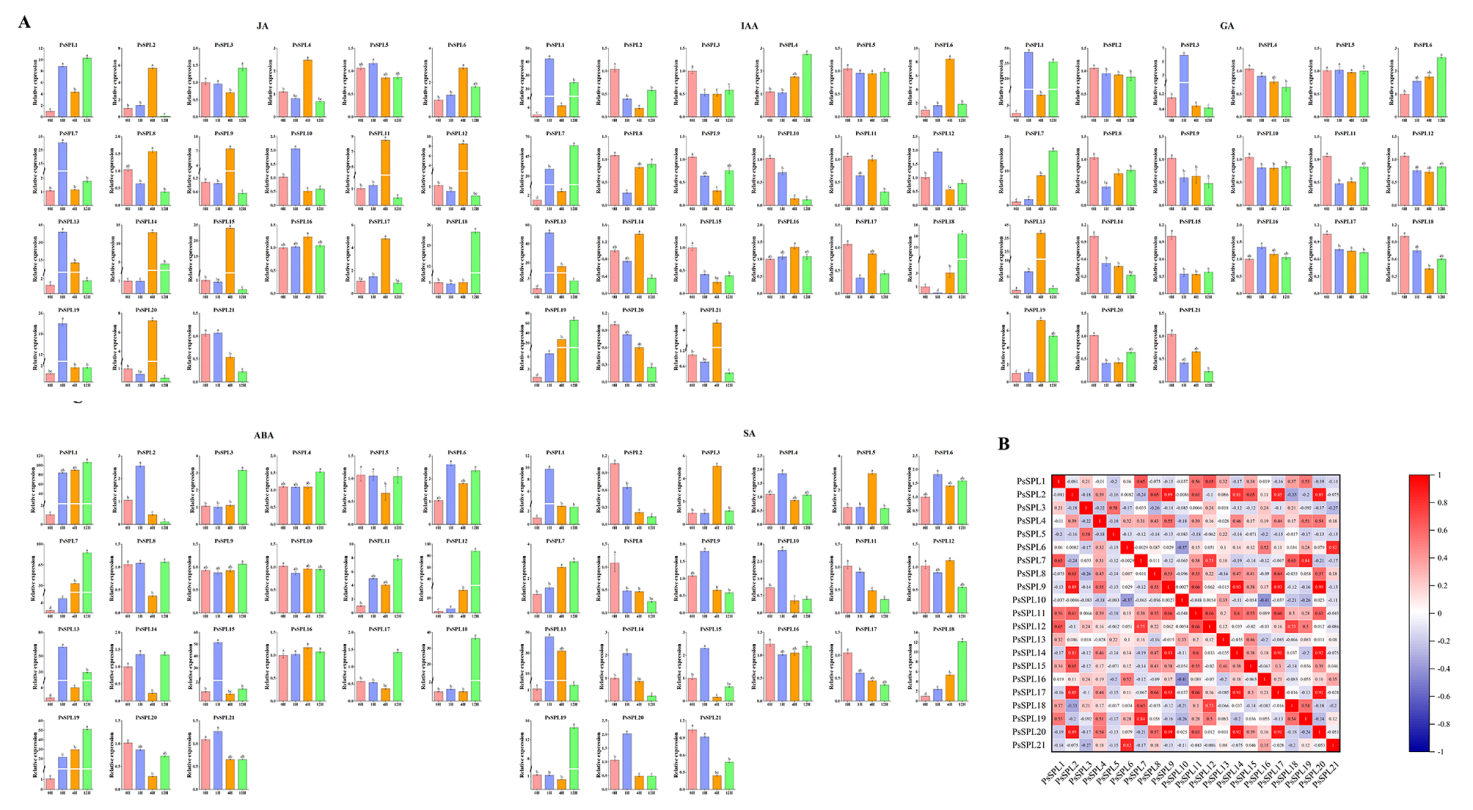
Expression analysis of 22 *PsSPL*s in fruits in response to different hormones (JA, ABA, IAA, SA and GA). (**A**) qRT-PCR was utilized to detect the expression patterns of 22 *PsSPLs*. Error bars (*n* = 3) represent the standard error. Lowercase letters above the bars indicate significant differences (*α* = 0.05, LSD) among treatments. (**B**) Coexpression analysis of 22 *PsSPL*s. Positive numbers = positive correlation; negative numbers = negative correlation. Red numbers indicate a significant correlation at the 0.05 level

Previous studies demonstrated that some *SPL* genes are involved in hormone regulation [49–51]. Therefore, we treated pea plants with five hormones (ABA, JA, SA, GA, and IAA) and evaluated the changes in the transcription levels SPL genes. The expression of most genes was upregulated under ABA, JA, SA, and IAA treatments, whereas most genes were downregulated under GA treatment (Fig. 9). *PsSPL1* showed the highest expression level under ABA and GA induction, whereas *PsSPL13* exhibited the highest expression level under SA and JA induction. *PsSPL19* had the highest expression level under IAA induction. Interestingly, these five hormones exhibited peak expression levels at different times. For example, most *PsSPLs* showed the highest expression levels at 12 h under ABA induction, 1 h under SA induction, and 4 h under JA induction. This finding implies that different hormones regulate the growth and development of pea plants by modulating the expression levels of *PsSPLs* at different times. We observed upregulation in the expression of *PsSPL5* under SA treatment, whereas the expression of this gene was downregulated under other hormone treatments.

## Discussion

Pea is a vegetable crop and an important legume crop [1]. which is rich in proteins, vitamins and minerals and is widely used in food processing and household consumption [2]. In agriculture production, peas are commonly used as green manure or feed plants to improve soil quality and can be rotated with other crops to increase agricultural yields [52]. Additionally, the pea genome is relatively small and the genetic background is relatively simple, so it is easy to study and manipulate [53, 54]. Therefore, peas are widely used as a model plant for plant genetics, biology and agricultural research. The *SPL* gene family plays an important role in fruit development [55–57]. Investigating the function and regulation mechanism of *SPL* gene family members during pea fruit development and abiotic stresses can make better understand the molecular mechanisms underlying pea pod formation. Furthermore, it provides a crucial theoretical basis for improving crop varieties and fruit yield and quality.

We identified 22 *SPLs* in the pea genome and explored the sequence and structural characteristics of these genes. The SPL Protein sizes range from 139 (*PsSPL*3) to 1025 (*PsSPL*9) amino acids. The maximum molecular weight of these proteins was 113.918 kDa (PsSPL18) and the minimum molecular weight was 15.9 kDa (PsSPL4). The number of introns in all *PsSPL*s ranges from 0 to 14. *PsSPL19* has a maximum of 14 introns, while *PsSPL5* has no intron structure. Introns play a key role in genome evolution by regulating gene diversity, gene expression regulation, and influencing protein diversity. The intron structure of subfamily II genes is the most complex, suggesting that subfamily II genes may have more functions than other genes. Intron-free genes usually exhibit higher transcription and translation efficiency than intron-containing genes. It is speculated that under stress conditions, the transcriptional regulation process of intron-free genes may be simpler and faster after responding to stimuli [58–60].

In this study, 22 *PsSPL*s and their homologous in *Arabidopsis thaliana* were clustered. The *SPLs* of both were found to be divided into eight subfamilies, each containing at least one *AtSPL* gene (Fig. 1). Obvious structural and functional differences may be structural and functional differences exists among different SPL subfamilies. There are four fragment repetition events in *PsSPL*, which may be generated during the evolution of *PsSPL* family [46, 47]. The diversity of sequence length within the *PsSPL* family may confer adaptability to different environmental and ecological conditions on peas, thus affecting the survival of pea species [61]. The *SPLs* clustering results of pea and other six plants showed that *PsSPLs* were highly homologous to soybean *SPLs* (Fig. 5), although there were obvious differences in morphology and growth habits between the two crops. Phylogenetic analysis shows that subfamily II members are very complex and contain all identified conserved motifs. As a binding protein to gene cis-elements, SPL plays a role as a key regulatory element [62]. The diversity of motifs may cause PsSPL to bind promoter cis-elements of multiple genes to regulate downstream gene expression in a complex way [63, 64], thus exerting diverse functions.

Gene expression analysis is often used for functional prediction to elucidate regulatory mechanisms and biological processes [65]. qRT-PCR analysis showed that most representative *PsSPLs* were highly expressed in stems, leaves and flowers, and were involved in the regulation of pod development in pea (Fig. 7). Rice *OsSPL4*, *OsSPL13* and *OsSPL16* promote the increase of rice grain width by regulating the level of cytokinin synthesis, and ultimately affect the grain morphological size [66–68], which seems to support the involvement of *SPLs* in seed or fruit formation. Under the four non-stress conditions, most of the genes of pea were induced to up-regulate, and the expressions of *PsSPL1*, *PsSPL5*, *PsSPL7* and *PsSPL18* of subfamily II were significantly up-regulated in stems under cold and heat stress (Fig. 8). It has been reported that *AtSPL1* and *AtSPL12*, members of II subfamily enhanced the heat resistance of inflorescence by regulating ABA signaling pathway, thereby reducing the sensitivity of flower organs to high temperature stress [69], suggesting that some *SPLs* may have similar regulatory mechanisms in the response of pea and *Arabidopsis thaliana* to heat stress. Moreover, miR156SPL can target *AtSPL9*, regulate the binding activity of *AtSPL9* with C-REPEAT binding factor 2(CBF2) and induce the expression of *AtSPL9* under cold stress, and enhance freezing resistance [70]. In apples, the miR156/ *MdSPL13* module regulates the salt tolerance of apples by targeting the promoter of *MdWRKY100*, and overexpression of *MdWRKY100* will enhance the salt tolerance of apples [71], indicating that miR156 usually mediates the response of *SPL* to salt stress and drought stress. In addition, OsSPL10 imparts drought resistance to rice by regulating the expression of *OsNAC2* [72], while *TaSPL6* plays a negative regulatory role in plant drought stress response by reducing the expression of some genes involved in stress response, and overexpression leads to increased sensitivity of wheat to drought stress [73]. In addition, we predict the cis-acting elements of the *PsSPLs* promoters in order to find transcription factors that can bind to them, which may be closely related to abiotic stress response (Additional file 6: Table S6). All the above studies provide research ideas for revealing the mechanism of *PsSPL* regulating abiotic stress response. Under four different plant hormone treatments, the expression of most *SPLs* was increased to varying degrees, mainly at 1 h, 4 h and 12 h after SA, JA and ABA hormone treatments, and the expression of *PsSPL1* was increased under ABA and GA induction (Fig. 9). Previous studies have shown that in rice, OsSPL12 can directly interact with nine proteins related to GA signal and participate in the regulation of GA synthesis in rice grains [49], providing clues and references for the mechanism by which *PsSPL* family participates in GA signal transduction pathway to regulate pod development, promote seed dormancy and inhibit ear germination, etc. *PsSPL13* is highly expressed by SA and JA. *PsSPL19* may be associated with disease resistance, while IAA induced high expression of *PSSPL19* which may promote pod cell elongation or fruit enlargement.

## Conclusion

In this study, we identified 22 *PsSPLs* in the pea genome and conducted a comprehensive analysis of the structure and potential functions of these genes. The 22 *PsSPLs* were unevenly distributed across seven chromosomes and were classified into eight subfamilies based on homology with *AtSPLs*. All *PsSPLs* harbored the SBP domain. Subfamily II exhibited the most complex intron-exon structure and had the highest number of motifs, indicating the functional diversity of genes in this group. The *PsSPLs* family in peas lacked tandem duplications, but segmental duplications were observed, implying that segmental duplication was involved in the evolution of the gene family. Phylogenetic analysis showed that *PsSPLs* were highly homologous to soybean *SPLs*. The expression profile analysis of *PsSPLs* indicated that these genes may play an important role in the pea growth and development, pod maturation and environmental response. In particular, *PsSPL19* is considered as a potential candidate gene for exploring pea breeding.

### Electronic supplementary material

Below is the link to the electronic supplementary material.

Supplementary Material 1

Supplementary Material 2

Supplementary Material 3

Supplementary Material 4

Supplementary Material 5

Supplementary Material 6

## Data Availability

Whole genome sequence information for *Pea* was obtained from the Ensembl genome website (http://ensemblgenomes.org). The seed used in this experiment was Zhongwan 6. The datasets supporting the conclusions of this study are included in the article and in additional files.

## Abbreviations

SPL: SQUAMOSA promoter-binding protein-like
*PsSPL*: Pisum sativum L. SPL
*q*RT-PCR: quantitative real-time polymerase chain reaction
AtSPL: Arabidopsis thaliana SPL
HMM: Hidden Markov Model
pI: isoelectric point
LG: linkage group
DPA: days post anthesis
